# A rat model of dual-flow liver machine perfusion system

**DOI:** 10.1590/acb387723

**Published:** 2023-10-30

**Authors:** Masayuki Ohara, Jun Ishikawa, Syuhei Yoshimoto, Yoji Hakamata, Eiji Kobayashi

**Affiliations:** 1 Nippon Veterinary and Life Science University – School of Veterinary Nursing and Technology – Tokyo, Japan.; 2 Screen Holdings Co., Ltd. – Innovation Development Department – Tokyo, Japan.; 3 Jikei University School of Medicine – Department of Kidney Regenerative Medicine – Kyoto, Japan.

**Keywords:** Oxygenation, Perfusion, Rats, Ischemia, Liver

## Abstract

**Purpose:**

As clinical liver perfusion systems use portal vein and artery flow, dual perfusion techniques are required even in small animal models in order to reproduce clinical setting. The aim of this study was to construct a new dual-flow perfusion system in rat model and optimized the oxygen supply to ensure the aerobic metabolization.

**Methods:**

The dual-flow circuit was fabricated using rat liver and whole blood samples as perfusates. The oxygen supply was controlled according to the amount of dissolved oxygen in the perfusate. Perfusate parameters and adenosine triphosphate (ATP) levels were analyzed to evaluate organ function and metabolic energy state. Stored whole blood also tested the suitability as perfusate.

**Results:**

Stored blood showed decrease oxygen delivery and liver function compared to fresh blood. Using fresh blood as perfusate with air only, the dissolved oxygen levels remained low and anaerobic metabolism increased. In contrast, with oxygen control at living body level, anaerobic metabolism was well suppressed, and tissue ATP content was increased.

**Conclusions:**

We developed a new dual-flow system that enable to reproduce the clinical settings. The perfusion system showed the possibility to improve the energy metabolic state of the perfused organ under appropriate partial pressure of oxygen.

## Introduction

To solve the problem of donor liver shortage, research and development of machine perfusion technology and its practical applications are progressing toward the utilization of marginal livers that have been considered unsuitable for transplantation. Owing to certain anatomical characteristics, livers must be perfused via the hepatic artery and portal vein, and normothermic machine perfusion devices used clinically for the liver use this dual flow^1^^-^ 3. Attempts are being made to develop applications for better resuscitation functions and perfusion methods for long-term culture of segmented livers. For clinical use, a perfusion device has been developed in a porcine liver model, and a machine perfusion model using a small-animal model has been used to screen drugs favorable for perfusion and to elucidate the mechanism of organ function^4^.

We previously developed a machine perfusion device for clinical use through a porcine liver model^5^; in this study, we developed a rat liver perfusion model to overcome challenges that may be encountered, for example, in the development of a new perfusate. A MEDLINE search of machine perfusion of the liver in rat models revealed 146 reports (as of November 9, 2021). Surprisingly, only eight articles reported simultaneous perfusion via the portal vein and hepatic artery^4^^,^ 6 ^-^ 12. It has long been reported that arterial reconstruction is unnecessary for rat liver transplantation models^13^; however, for ex-vivo machine perfusion in small-animal models, the necessity of hepatic arterial perfusion is unknown.

In an effort to perfuse small animals in an environment similar to that of the living body, we constructed a whole-blood perfusion system under normal temperature that can perfuse the portal vein and hepatic artery simultaneously. We also evaluated the availability of fresh blood immediately after collection and stored blood mixed with the citrate-phosphate-dextrose with adenine (CPDA) solution as the oxygen carrier fluid for perfusion.

Next, based on the lack of clarity regarding the appropriate oxygen concentration of the perfusate^14^, we developed a mechanism to control the amount of dissolved oxygen during live extracorporeal perfusion and analyzed the effect of oxygen concentration on the explanted liver. In addition, using a luciferin-luciferase luminescence system that can visualize adenosine triphosphate (ATP) in organs^15^^,^ 16, we continuously measured the amount of ATP in organs during ex-vivo perfusion and evaluated the metabolic state of the liver.

## Methods

### Animals

Male Lewis rats (250–350 g) and luciferase-expressing transgenic Lewis rats (250–320 g)^15^ were used. The experiments were approved by the Ethics Committee of Nippon Veterinary and Life Science University (Permit No. 2021S-14). All animal experiments were performed under inhaled isoflurane anesthesia (Pfizer Inc., New York, NY, United States of America).

### Hepatectomy procedure

After transverse laparotomy, the ligament around the liver was dissected. The hepatoesophageal vascular plexus was then ligated and dissected. Subsequently, the common bile duct was cannulated using a 24-gauge catheter (indwelling needles; Terumo Corporation, Tokyo, Japan). The esophagus was ligated using a 4-0 silk thread (Akiyama-Seisakusyo Co., Ltd., Tokyo, Japan), and the pharyngeal side was dissected. The common hepatic artery was exposed by moving the stomach and spleen to the left. After ligating the gastroduodenal artery, splenic vein, left gastric artery, and splenic artery, the distal portion was excised. After the portal vein was exposed, 0.3 mL of 1,000 U/mL heparin (Nipro Corporation, Osaka, Japan) was administered through the inferior vena cava (IVC). A 16-gauge catheter was then inserted into the portal vein and perfused with Ringer’s solution. In addition, the IVC of the liver was quickly dissected to allow for perfusate drainage.

The aorta was exposed, and a 4-0 silk thread was applied to the side of the heart from the bifurcation of the common hepatic artery. A 22-gauge catheter (indwelling needles; Terumo) was inserted into the aorta and ligated. The 4-0 silk thread on the cardiac side was ligated, and the aorta was separated. After confirmation of liver perfusion with Ringer’s solution, the suprahepatic IVC and surrounding connective tissue were dissected, and the liver was removed. Under the donation-after-circulatory-death (DCD) protocol, an incision was made between the ribs, and the thoracic aorta was blocked using forceps before excision. After confirming that the heartbeat had stopped, the animal was left for 60 min before perfusion with Ringer’s solution.

### Blood collection for perfusion

We analyzed the amount of blood collected from the IVC alone and from the IVC and aorta. In the group with only intravenous blood sampling, a transverse incision was made in the abdomen, and blood was collected from the IVC using a 21-gauge needle (Terumo). After respiratory arrest, blood collection was continued via the IVC, and cardiopulmonary massage was performed until no more blood was collected. In the combined aortic sampling group, after confirming respiratory arrest due to hypotension caused by venous blood sampling, blood was collected from the abdominal aorta using the same syringe while performing cardiopulmonary massage.

For stored blood experiments, the blood was collected the day before the perfusion experiment, and 80 mL of whole blood was mixed with 11.2 mL of CPDA solution (Kawasumi Laboratories, Inc., Tokyo, Japan) and kept refrigerated. Because of the time required to collect fresh blood on the day of the experiment, stored blood was also used, as other individuals had to be sacrificed to operate the perfusion circuit. Stored blood was limited to a few days in which the blood composition was unchanged, considering platelet longevity and other factors, as in human clinical practice. Fresh blood was collected immediately before the perfusion experiment, and 80 mL of whole blood mixed with 3 mL of 1,000 U/mL sodium heparin (Nipro) was used as the perfusate.

### Use of blood for perfusion using the in-vivo dilution method

Mature male Lewis rats (body weight, 380–540 g; mean, 463.8 ± 88.8 g; n = 4) were used. A transverse incision was made in the abdomen, and blood (0.5 mL) was drawn from the IVC under anesthesia. The same area was then punctured with a 16-gauge SURFLO needle, the needle was fixed to the abdominal wall with instant adhesive, and a rapid infusion tube (SF-ET2022L; Terumo) was placed. While observing respiration and heart rate, 25–40 mL (31.25 ± 6.3 mL) of warm plasma substitute (Voluven 6% solution; Otsuka Pharmaceutical Factory, Inc., Tokushima, Japan) was infused. Following respiratory arrest, the infusion was stopped for a few seconds, and the animal was allowed to recover spontaneous breathing. Approximately 5 min after starting the Voluven infusion, blood was collected from the same site^17^. Blood collection was terminated when the rats stopped breathing.

The hemodilution rate was determined as the dilution rate using the hematocrit values of the blood before treatment and of the collected blood after treatment. As a control for the blood obtained by in-vivo dilution, blood from untreated rats was prepared by adding boluses to obtain comparable hematocrit values in vitro.

### Perfusion circuit

**Figure 1:**
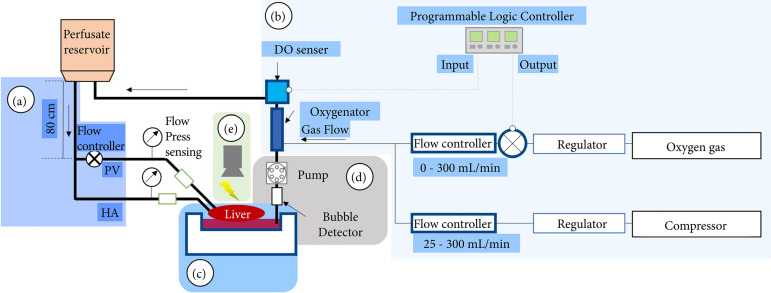
Schematic image of continuous flow perfusion circuit using oxygen control units. This circuit employs a **(a)** hydraulic head difference flow for the portal vein and hepatic artery. Portal flow was regulated by a flow controller to prevent excess flow and pressure. The perfusate was accumulated in the **(b)** chamber and drained using a pump assembled with a **(c)** bubble detector to recirculate to a reservoir. The concentration of dissolved oxygen was controlled by a programmable logic controller that enabled switching of the oxygen gas bulb owing to the dissolved oxygen value sensed by the **(d)** sensor. Luminescence of the liver was detected using a **(e)** luciferase imaging system during perfusion.

Source: Elaborated by the authors.

We adopted a semi-closed system in which the liver received continuous flow via the portal vein and hepatic artery using a hydraulic head difference, and the perfusate into the chamber was drained using a pump (TP-1973D; AS ONE Corporation, Osaka, Japan) (Fig. 1). In addition, a bubble detector (FD-XS1; Keyence, Osaka, Japan) was attached to the recovery circuit. To prevent excessive draining, the drain pump was controlled according to the detection of bubbles entering the circuit due to the drop in liquid level in the chamber. This system allowed the liver to flow out of the perfusate in a semi-floating state^17^.

The temperature was controlled at 37°C by a combination of a temperature controller and a warm bath while measuring the temperature in the circuit with a thermometer. The flow rate was controlled by adjusting the drip rate with a creme and measuring the flow rate with a flowmeter. The pressure and flow rate of the perfusate flowing into the liver during continuous perfusion were measured using a pressure gauge (ZSE80F; SMC Corporation, Tokyo, Japan) and a flow meter (FD-XS1; Keyence), respectively. The perfusate was sampled hourly and analyzed using a blood analyzer (i-STAT 1; Abbott Laboratories, Abbott Park, IL, United States of America). Secreted bile was collected hourly via a cannula into a 1.5-mL Eppendorf tube.

### Oxygen control mechanism

The perfusate was oxygenated while supplying gas via an oxygen exchanger (M60-A; Nagayanagi Co., Ltd., Tokyo, Japan). The partial pressure of oxygen (pO_2_) in the liquid was measured using a dissolved oxygen meter (VisiFerm DO Arc 120 H0; Hamilton Co., Reno, NV, United States of America). Depending on the measured values, three types of gas supply were prepared and made available: pure oxygen, air using a compressor, and pure oxygen and air that could be mixed as desired.

### Study design

**Figure 2:**
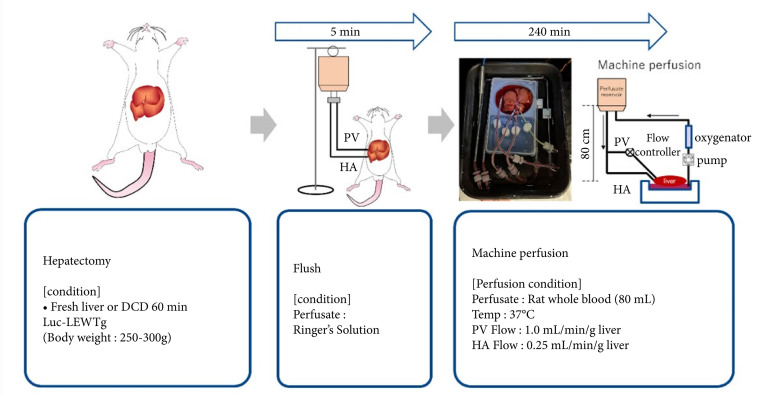
Experimental protocols and perfusion condition. Livers were removed and perfused with Ringer’s solution or rat whole blood at 37°C. The perfusate flow rate was set at 1 or 0.25 mL/min/g liver.

Source: Elaborated by the authors.

Using the perfusion circuit, experiments were performed using the protocol as depicted in Fig. 2. First, fresh livers were removed from mature wild-type Lewis rats and luciferase-expressing transgenic Lewis rats. Ringer’s solution (Otsuka Pharmaceutical Factory) was infused through the portal vein and hepatic artery. Next, the liver was reconnected to the perfusion circuit and perfused with 100 mL of Lewis rat whole blood for 4 hours at 37°C. The height of the reservoir was set at 80 cm from the liver to deliver 0.25 mL/min per gram of liver to the hepatic artery. On the portal vein side, the flow rate was maintained at 1 mL/minper gram of liver using a cremulator to prevent the flow rate from increasing because of the hydraulic head difference.

### Luciferin assays

Thirty microliters of 30 mg/mL D-luciferin (AAT Bioquest, Inc., Sunnyvale, CA, United States of America) diluted in saline was administered through the injection port of the portal inflow, and luminescence intensity was measured while maintaining perfusion and exposure for 60 seconds with an imaging system (NightOWL II LB983; Berthold Technologies GmbH & Co. KG; Baden-Württemberg, Germany).

### Statistical analysis

All experimental data are presented as the mean ± standard deviation. Error bars are standard errors unless otherwise stated. The experimental data were subjected to hypothesis testing using the Student’s t-test. The significance level was set at P < 0.05.

## Results

### Hepatectomy for dual flow

**Figure 3:**
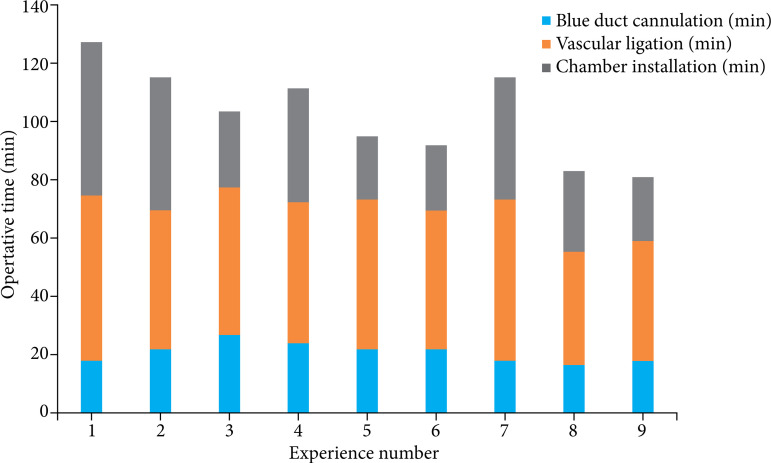
The required time of operative procedures. Each colored bar represents the time from laparotomy to bile duct cannulation (blue), vascular ligation before liver extraction (orange), and chamber installation to perform ex vivo circulation (gray).

Source: Elaborated by the authors.

The time from liver removal to installation in the perfusion chamber followed a learning curve (Fig. 3). The time used to cannulate the bile duct and to remove the liver did not decrease with the number of experiments; however, the time used to place the liver in the chamber was 22 min in experiment 9 compared with 53 min in experiment 1. This was because all branches around the aorta were ligated beforehand during installation in the chamber, which reduced the time needed to determine the leakage of perfusate from the artery.

### Comparison of fresh blood, stored blood, and diluted blood as perfusate

Because the circuit requires a volume of approximately 80 mL, we examined the collection route and the possibility of using stored and diluted blood to efficiently collect blood to use as the perfusate. To investigate the possibility of using diluted blood, we performed an in-situ dilution (ISD) method, in which blood was collected from rats while the plasma substitute was injected into the body, and the properties of the collected blood were analyzed^17^. In the ISD group, in which blood was diluted in vivo by a one-shot bolus of plasma substitute, the amount of blood collected was three times that of the ordinary blood collected from the IVC, and the hematocrit value decreased to one-third, indicating that the plasma was diluted three times in vivo.

Compared with the in-vitro dilution (IVD) group, in which blood taken from the IVC was diluted three times with substitute plasma in vitro, the concentrations of inorganic ions and biologically derived substances such as glucose, lactate, and blood urea nitrogen (BUN) in the ISD group remained similar to those of physiological whole blood. In the IVD group, pH was not significantly different from physiological whole blood; however, in the ISD group, the pCO_2_, lactate, K, glucose, BUN, creatinine, and anion gap values were not significantly different. This suggests that, in the ISD group, these small molecules were rapidly transferred from biological tissues into the blood, and the values were similar to those of biological blood. In the IVD group, oxygen gas parameter values (oxygen saturation [sO_2_] and pO_2_) deviated greatly from biological values, suggesting that the values increased due to rapid oxygen dissolution from room air and the plasma substitute during IVD.

In this study, blood collected by the ISD method was not used as a perfusate because it was thought that the oxygen supply environment was not equivalent to that of a living organism because of the decrease in the hematocrit value; however, this method is considered effective in identifying blood factors that need correction to improve the perfusate.

Next, we examined the amount of blood that could be collected from each route. Using 350 ± 10 g rats, the blood volume obtained from the venous route alone was 10.7 ± 1.25 mL (n = 3), and the blood volume obtained from the combined venous and arterial routes was 12.8 ± 0.65 mL (n = 3)^17^. Therefore, in subsequent experiments, we used both the venous and arterial routes for blood collection.

**Figure 4:**
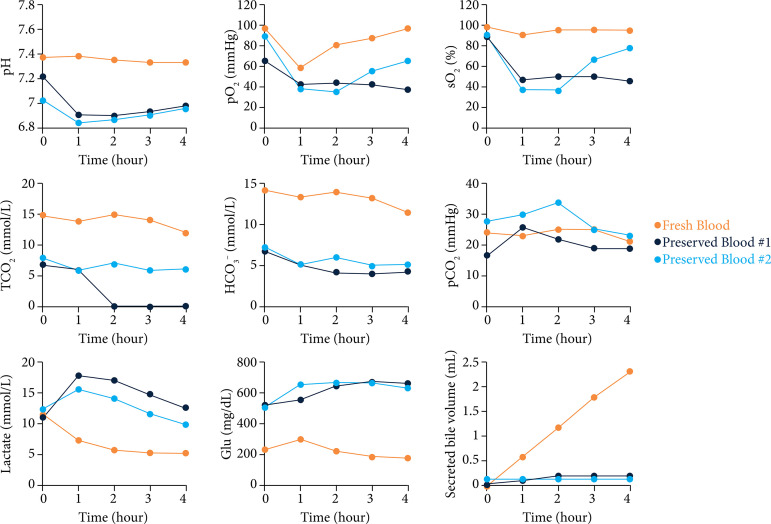
Perfusate analysis during extracorporeal perfusion using stored blood. Each figure shows the blood gas parameters, glycolytic substrates, and secreted bile volumes. These experiments were performed using only air supplementation (100–200 mL/min) and non-diluted fresh blood (n = 1, orange) or stored blood (n = 2, purple and light purple).

Source: Elaborated by the authors.

The blood-gas parameters during perfusion and the analysis of each blood component after 4 hours of perfusion with stored blood are shown in Fig. 4. At the start of perfusion, the stored blood group values differed from those of the fresh blood group due to the pH and glucose of the CPDA solution. The pO_2_ in the perfusate was higher in fresh blood, whereas the sO_2_ was clearly lower in the stored blood. Additionally, HCO_3_^—^ and total CO_2_ values were decreased in the stored blood compared to those values in the fresh blood, suggesting that the oxygen-binding capacity of erythrocytes decreased in stored blood and aerobic metabolism decreased due to reduced oxygen-carrying capacity. This was also suggested by the lactate levels being higher in the stored blood. Furthermore, bile secretion was nearly absent in the stored blood group.

Based on the described results, we decided to use non-diluted fresh blood collected from six adult male rats as the perfusate in this study, as its function as an oxygen carrier was not reduced.

### Investigation of oxygenation conditions by machine perfusion

**Figure 5:**
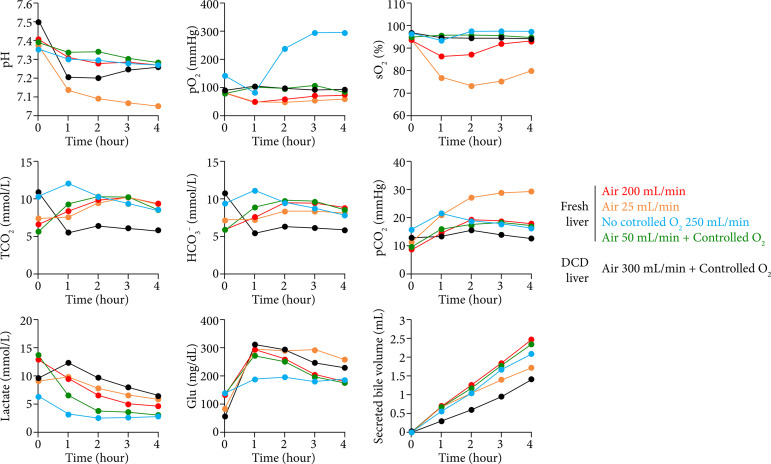
Perfusate analysis during extracorporeal perfusion under a controlled gas supply. Each figure shows the blood gas parameters, glycolytic substrates, and secreted bile volumes during the perfusion of fresh livers and donation-after-circulatory-death (DCD) livers using non-diluted fresh blood. The conditions of gas supplementation were as follows: fresh liver, only air at 200 mL/min (n = 1, red); only air at 25 mL/min (n = 1, yellow); only O_2_ gas with a continuous flow of 250 mL/min (n = 1, blue); air at 50 mL/min; and controlled O_2_ supplementation (n = 1, green). The parameters using DCD livers are shown with air at 300 mL/min and controlled O_2_ supplementation (n = 1, black).

Source: Elaborated by the authors.

**Figure 6:**
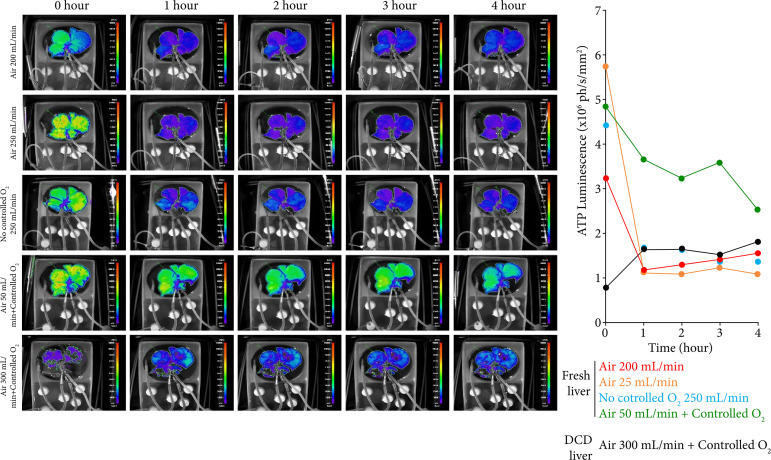
Adenosine triphosphate luminescence of luciferase-expressing transgenic rat livers. Each image represents luminescence of the liver during perfusion for 4 hours. The graph on the right shows the quantitative values of luminescence for each sample and time point. The conditions of gas supplementation were as follows: fresh liver, only air at 200 mL/min (n = 1, red); only air at 25 mL/min (n = 1, yellow); only O_2_ gas with a continuous flow of 250 mL/min (n = 1, blue); air at 50 mL/min; and controlled O_2_ supplementation (n = 1, green). The parameters using donation-after-circulatory-death livers are also shown with air at 300 mL/min and controlled O_2_ supplementation (n = 1, black).

Source: Elaborated by the authors.

The perfusate and circulation parameters and bile production from the liver during machine perfusion with non-diluted blood for 4 hours are shown in Fig. 5. To evaluate the gas supply condition, we employed several air and oxygen gas flow combinations that ensured appropriate sO_2_ of red blood cells and pO_2_ in the perfusate. With pure oxygen only, the pO_2_ increased to > 300 mmHg, whereas with air only the dissolved oxygen level decreased and remained low from the start of perfusion. In contrast, the dissolved oxygen was maintained between 80 and 100 mmHg under controlled conditions, even when the air flow was 50 mL/min. Furthermore, the sO_2_ of red blood cells could be maintained at 95% or higher by adding oxygen. In addition, the total CO_2_ and HCO_3_^-^ levels were lower in the DCD liver, indicating reduced aerobic respiration. Rapid acidosis was observed under low-gas-supply conditions in the DCD liver. The lactic acid concentration was greatly reduced by the addition of oxygen, and the addition of air tended to result in a higher lactic acid concentration than that from adding oxygen. Luciferin luminescence decreased rapidly to > 25% of the initial perfusion level after 1 hour of perfusion in the absence of dissolved oxygen control and remained constant thereafter. In the DCD liver, luminescence remained at the same level thereafter, although ATP recovery by perfusion was observed (Fig. 6). In contrast, no significant decrease was observed by controlling the dissolved oxygen, and it was confirmed that the luminous intensity was higher than that of the noncontrolled group, even after 4 hours of perfusion. These results indicate that, by controlling the dissolved oxygen concentration, an appropriate aerobic respiratory rate and high level of energy production in the tissue could be maintained.

## Discussion

This study clarified the necessity of controlling the oxygen concentration during ex-vivo machine perfusion and showed that ex-vivo perfusion with appropriate oxygen control could maintain energy production. Furthermore, construction of a perfusion system that reflects the blood circulation pathway of the body using whole blood will play an important role in the development of perfusates in the future. This system also verifies the necessity of arterial perfusion in an environment similar to biological conditions.

Oxygen is essential for aerobic respiration, enabling organisms to produce energy more efficiently. Oxygen bound to red blood cells is supplied to tissues, whereas free oxygen is maintained at a low concentration in vivo. Adversely, excess oxygen causes an increase in reactive oxygen species, peroxidation of cell membranes, and a decrease in protoplasmic flow^18^^-^ 20. Physiologically, hyperoxia during cardiopulmonary bypass decreases the deformability of red blood cells, leading to peripheral circulatory disturbances and postoperative organ dysfunction^21^.

In this study, the amount of ATP in tissues decreased under hyperoxic conditions, and it is clear that an inappropriate hyperoxic environment can be a factor in organ damage. Although no statistical determination due to the number of individuals in the ATP visualization system, these results suggest the need for an ex-vivo perfusion system that maintains an appropriate oxygen concentration and demonstrate the effectiveness of perfusion devices that reflect the biological environment.

The liver has a specific vascular pathway, which is the proper hepatic artery (a nutrient vessel) and the portal vein (a functional vessel), that differs from the simple arterial-venous pathway. The importance of the arterial route has long been recognized, especially in the extrahepatic bile duct, which receives a blood supply only from the artery, and it has been shown in clinical practice that arterial back-table perfusion of the graft can inhibit bile duct injury^22^. It is difficult to discuss the necessity of arterial perfusion in the ex-vivo perfusion model of small animals because most of the previous reports used only the portal vein as the perfusion vessel^23^. Although an orthotopic transplantation method of portal vein reconstruction alone showed good survival^13^, angiogenesis in the arterial pathway may have occurred after transplantation.

Liver transplantation models in rats and mice have a long history. In contrast to humans, they survive without hepatic artery reconstruction, and, due to the difficulty of surgical techniques, most experiments using perfusion have been conducted with blood flow in the portal vein only^23^. A few previous reports have utilized circuits capable of perfusing both the hepatic artery and portal vein^4^^,^ 6 ^-^ 12. Although this report compared dual and single perfusion and found no definitive differences, the need for an arterial pathway in a bio-reflective environment remains unclear because the perfusate used blood diluted with a medium, and pressure was not verified. Blood was used as the perfusate, and a dual perfusion system that simulates the living body was constructed. By reconfiguring this perfusion circuit, the effectiveness of arterial perfusion and arterial-only oxygenation in the future can be verified. This study provides a useful model that can be used as a basic technology for evaluation in a physiological environment.

Various metabolic reactions occur in living organisms, and, by switching metabolic pathways according to the surrounding environment, stable energy production is made possible^24^. Furthermore, not only changes in metabolic substrates but also mechanical effects can alter physiological functions, especially in vascular endothelial cells, which are strongly affected by mechanical effects^25^^,^ 26. Because cells respond to changes in the surrounding factors, it is necessary to consider these factors when setting up experimental conditions. In previous reports, various perfusion conditions have been employed, and the wide range of temperatures, perfusion rates, and perfusate compositions that govern metabolic activity make it difficult to uniquely compare the effects of specific factors on perfusion^27^.

A decrease in temperature and oxygen concentration leads to an increase in glycolysis^28^, and our study also showed an enhancement of lactate production under hypoxic conditions. In addition, when diluted blood is used as the perfusate, reduction in shear stress and oxygen supply due to a decrease in red blood cell concentration is expected. Therefore, it is necessary to establish more universal perfusion solutions and conditions that enable the expression of metabolic activities similar to those in vivo. The results of this research will contribute to the development of a perfusion solution based on our pseudo-living body technology and to the development of a resuscitation technology that can recover damaged organs.

## Conclusion

The system we developed is equipped with a unit that can supply the appropriate amount of oxygen and shows the need for optimizing the oxygen supply in ex-vivo perfusion using a small-animal model. In addition, the energy state of luciferase-expressing animals was analyzed in a perfusion environment mimicking the physiological state.

This study proposed a significant perfusion system that resolves the discrepancy between the small-animal model used in many basic studies and the large-animal model used in clinical studies. This perfusion system will strongly support small-animal studies for screening clinical drugs or novel organ preservation methods because it reflects the physiological state of the perfusate and clinical organ preservation devices. In the future, while screening perfusion components, we would like to clarify the effects of improving energy metabolism. We hope that this technology can be applied to systems used in large animals and in clinical settings to analyze metabolic mechanisms in small-animal models.

## Data Availability

Supplemental data

**Figure:** 

are available at https://doi.org/10.5281/zenodo.10035690.
