# Supplementary material for: A rat model of dual-flow liver machine perfusion system
Source: Acta Cir Bras. 2023 Oct 30;38:e387723. doi: 10.1590/acb387723 (PMC10664844; doi:10.1590/acb387723)
Supplement: Supplementary file 1 [file 1678-2674-acb-38-e387723-suppl1.pdf]

Supplemental figures

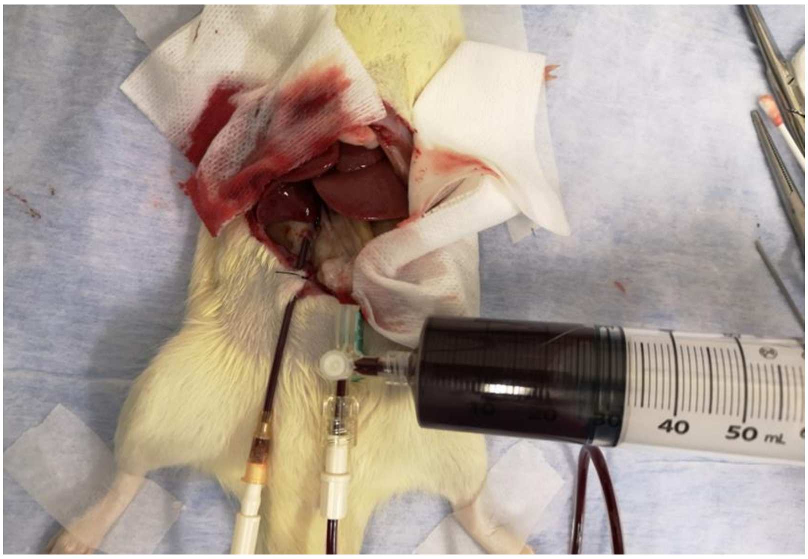

Supplementary Figure 1 - The image of bolus injection of plasma substitute. The bolus injection of plasma substitute was performed via inferior vena cava, and the blood was collected from same site. This technique allowed to collect the massive volume of blood from animals.

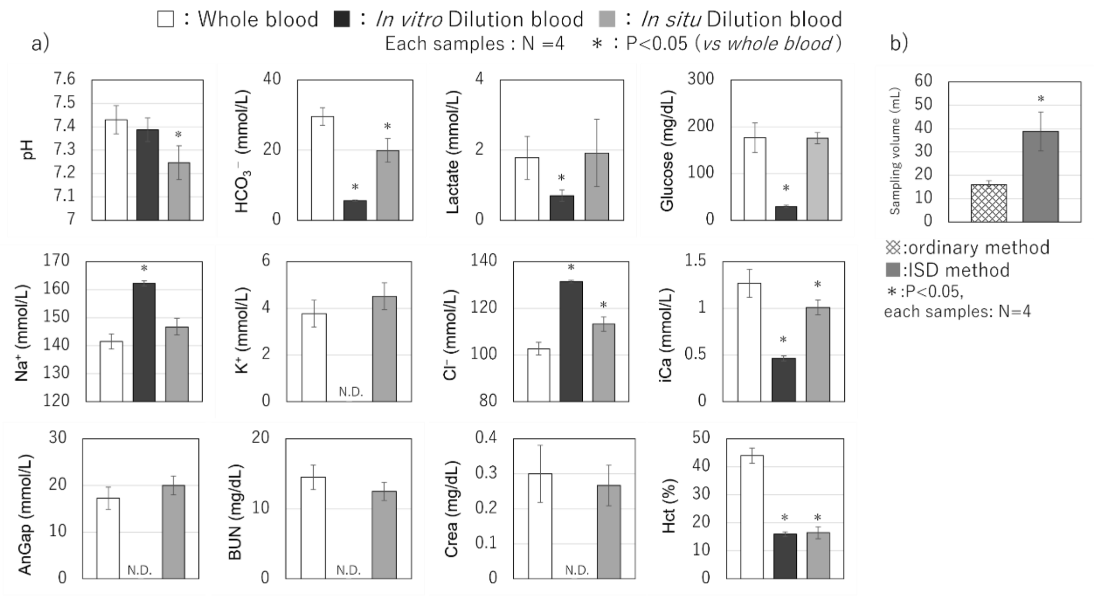

N.D.: value not detected; *P < 0.05.

Supplementary Figure 2 - The statistical analysis of blood parameters in each blood. (a) Blood parameters with sampling by each method. The statistical analysis was performed in each sample vs. whole blood. The colored bars represented whole blood (white); in-vitro dilution blood (black); in-situ dilution blood (grey). (b) The blood sampling volume of ordinary blood sampling via inferior vena cava (white), in-situ dilution method (grey).

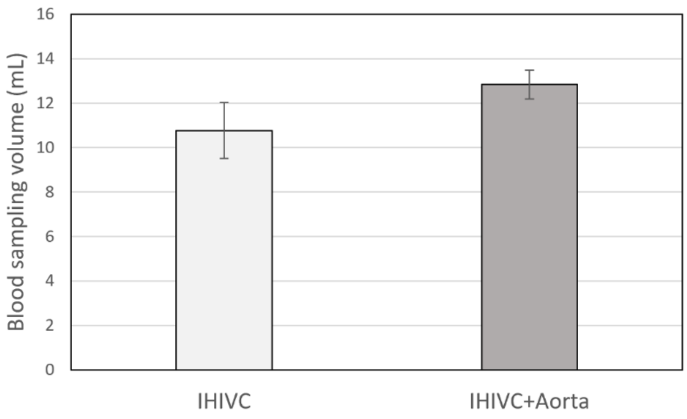

Supplementary Figure 3 - The comparative analysis of blood sampling volume in each collection route.

Source: All supplemental data were made by Ohara M.
